# 3D-Printed Teeth in Endodontics: Why, How, Problems and Future—A Narrative Review

**DOI:** 10.3390/ijerph19137966

**Published:** 2022-06-29

**Authors:** Tiago Reis, Cláudia Barbosa, Margarida Franco, Catarina Baptista, Nuno Alves, Pablo Castelo-Baz, José Martin-Cruces, Benjamín Martin-Biedma

**Affiliations:** 1 Department of Surgery and Medical-Surgical Specialties, School of Medicine and Dentistry, University of Santiago de Compostela, 15701 Santiago de Compostela, Spain; pablocastelobaz@hotmail.com (P.C.-B.); pepe3214@gmail.com (J.M.-C.); b.martinbiedma@gmail.com (B.M.-B.); 2 Faculty of Health Sciences, University Fernando Pessoa, 4249-004 Porto, Portugal; 3 FP-I3ID, Faculty of Health Sciences, University Fernando Pessoa, 4249-004 Porto, Portugal; cbarbosa@ufp.edu.pt; 4 CDRSP, Polytechnic Institute of Leiria, 2430-028 Marinha Grande, Portugal; margarida.franco@ipleiria.pt (M.F.); catarina.baptista@ipleiria.pt (C.B.); nuno.alves@ipleiria.pt (N.A.)

**Keywords:** endodontics, printing, three dimensional, tooth, 3D-printed teeth, review

## Abstract

Three-dimensional printing offers possibilities for the development of new models in endodontics. Numerous studies have used 3D-printed teeth; however, protocols for the standardization of studies still need to be developed. Another problem with 3D-printed teeth is the different areas of literature requested to understand the processes. This review aims to gather evidence about 3D-printed teeth on the following aspects: (1) why they are advantageous; (2) how they are manufactured; (3) problems they present; and (4) future research topics. Natural teeth are still the standard practice in ex vivo studies and pre-clinical courses, but they have several drawbacks. Printed teeth may overcome all limitations of natural teeth. Printing technology relies on 3D data and post-processing tools to form a 3D model, ultimately generating a prototype using 3D printers. The major concerns with 3D-printed teeth are the resin hardness and printing accuracy of the canal anatomy. Guidance is presented for future studies to solve the problems of 3D-printed teeth and develop well-established protocols, for the standardization of methods to be achieved. In the future, 3D-printed teeth have the possibility to become the gold standard in ex vivo studies and endodontic training.

## 1. Introduction

Bacteria are the main cause of pulpal and periapical pathologies. Therefore, all procedures used in endodontics are aimed at eliminating pulp tissue as well as many bacteria as possible [1,2].

The American Association of Endodontists defines root canal preparation as “Procedures involved in cleaning and shaping the canal system prior to obturation”, distinguishing between “biomechanical preparation” as the “use of rotary/reciprocating and/or hand instruments to expose, clean, enlarge and shape the pulp canal space, usually in conjunction with irrigants” and “chemomechanical preparation” as the “use of chemicals for irrigation of the root canal, demineralization of dentin, dissolution of pulp tissue and neutralization of bacterial products and toxins; used in conjunction with biomechanical preparation” [3].

To achieve these goals, a variety of manual rotary instruments have been proposed. The revolution in the manufacturing root canal instruments leads to a great variety of these endodontic instruments when comparing the design, alloys and type of movement [2,4,5,6,7,8].

Adequate knowledge of the shaping properties and performance of rotary or reciprocating instruments is essential to assist the clinician in selecting the appropriate system for different clinical situations, as new instrumentation systems continue to be introduced into the market [9,10].

For a successful root canal treatment, the knowledge of root canal anatomy is a basic requirement [11]. The general root canal anatomy of a normal tooth and other anatomic variations have been identified in many morphologic studies [11,12,13]; however, the anatomy of the root canal can vary widely at different levels of the root [14].

Teaching root canal treatment is challenging in undergraduate and postgraduate education. European Society of Endodontology guidelines state that students should be suitably trained to perform good-quality root canal treatment [15,16]. Nonetheless, a recent study stated that Spanish dental students treated simple clinical cases in 100% of the schools, but moderate clinical cases only in 40%, and only 60% of schools have minimum requirements for the number of root canal treatment cases [17]. Furthermore, in another study, a high proportion of final-year dental students were not confident in performing root canal treatment on multirooted posterior teeth [18]. Thus, in dental education, there is a need for hands-on pre-clinical training before a patient’s treatment is carried out for the first time [16,19,20,21,22].

Traditionally, for ex vivo studies and pre-clinical courses, extracted human teeth have been the standard practice [23,24,25,26,27,28] and are considered the best for simulating the clinical setting, despite the lack of evidence to support this claim [29], providing a good understanding of the internal anatomy and allowing students to experience the tactile sensation of working on dentine [30].

However, extracted human teeth has several drawbacks, which are being discussed in recent years: they are difficult to collect, there are ethical considerations, potential cross-infection risks and storage drawbacks and standardization issues [19,23,26,31,32,33].

Artificial teeth that reproduce the features of natural teeth may overcome these limitations [19,28,31,34]. In recent years, different factory-manufactured models have been introduced in the market, which are realistic and standardized. However, their costs are high, there is a limited selection of tooth types, the delivery times must be considered with a dependency on a manufacturer and the differences between the manufacturing process and the material used by each commercial brand are widely different [23,26,31,35].

Three-dimensional printing is a rapidly developing technology that has gained widespread acceptance in dentistry [36], and with costs constantly declining and more materials available, 3D-printed teeth offer unexpected possibilities for the development of new, individual models that are not yet available on the market or are too expensive to purchase in high quantities [26,28,31,34]. Nonetheless, the major criticism of 3D-printed teeth is the difference in radiopacity and hardness between resin and human dentine [19,23,31].

Numerous studies are using 3D-printed teeth; however, protocols for the standardization of studies still need to be developed since there exists a great methodological variation between these studies [23,24].

Another problem with 3D-printed teeth is the different and scattered areas of the literature, which need to be mastered to understand all the processes of printing them.

Hence, the aim of this review is to gather evidence on 3D-printed teeth on the following aspects: (1) why they are advantageous, pointing out the problems with natural teeth that they overcome; (2) how they are manufactured, describing all the phases and options of printing; (3) the problems and disadvantages that they present; and (4) future research topics to overcome these problems.

For the purposes of the present review, a search was conducted in PubMed, MEDLINE, Scopus, Web of Science, until 16 April 2022, using the keywords Endodontics, 3D printed teeth, artificial teeth, 3D printing, dentin, teaching, alone and with the Boolean operator “AND” in different associations. Original research studies, review articles and case reports, in English, without time limitation, were selected if relevant to the aims of this review. Additionally, cross references were used.

## 2. Why

Natural teeth are still the standard practice in ex vivo studies and pre-clinical courses [23,24,25,26,27,28]. Their advantages include natural tissue hardness, morphology, color, texture and radiodensity [24,28], but as listed before, they have several drawbacks:

(1) Difficult to collect: Increasing challenges in sourcing sufficient suitable natural teeth, probably as a result of improvement in health standards; it is time-consuming and requires a large number of teeth (wisdom teeth, teeth with highly complex root canal morphology and with previous root canal treatment are not acceptable, and natural teeth must present complete root development, an intact crown and intact roots) [19,23,24,27,30,35,37]. Nowadays, the biobank is considered a primary resource for the development of precision medicine. The purpose of biobanks is to support and improve clinical and biomedical research, especially for preclinical trials [38,39]. As a result, these teeth are not available for teaching purposes.
(2) Ethical considerations: Previous owners of natural teeth, representing biomaterial, should have declared their consent to the use of their teeth [23,28,32]. A worrying phenomenon is that in buying human teeth online, pressures to source teeth are likely to encourage investigators and students not to question where such teeth are from and why they have been extracted [32].
(3) Potential cross-infection risk and storage: Natural teeth are grossly contaminated, difficult to sterilize and common disinfection procedures, such as using sodium hypochlorite and hydrogen peroxide, are not effective [40,41]; teeth could also be damaged or altered by the sterilization and storage procedures. In a study that investigated the effect of disinfection methods on tooth resistance to fracture, it was found that autoclaving teeth reduced their resistance to fracture [42]. Another study investigated the effect of storage conditions on tooth resistance to fracture, and it was found that teeth stored in water have a higher resistance to fracture than teeth stored dry [43], so the preparation of natural teeth is an unpleasant task as they must be stored in a strong-smelling liquid [44].
(4) Standardization: The difficulty of creating well-balanced groups in ex vivo studies and an equal student assessment is a consequence of the intricate anatomy of the root canal system [14,25,26,31,33,45]. As a consequence, studies might demonstrate the effect of canal anatomy rather than the variable of interest [33].

Students normally comment that the anatomical variability does not allow a valid assessment of their individual performance [37].

The most common method used in ex vivo studies is to create pair-matched tooth samples according to their anatomical features, such as the tooth type, root length, root anatomy, degree and angle of curvature and root canal diameter, or even with paired contralateral teeth. Some studies allocated teeth from the same group by randomization with or without radiographs [23,25,46].

Recently, some studies started to use specific anatomical parameters identified by means of a microcomputed tomography (micro-CT) scanning [23,25]. De-Deus et al. 2020 compared the randomized method, radiographic method and micro-CT method. It was concluded that the micro-CT method was able to provide better control of the anatomical variance confounding effect. Nonetheless, this study was carried out from an initial pool of 1708 single-rooted mandibular incisors, and for the group of the micro-CT method, 251 teeth were chosen, which were scanned, to create two anatomically pair-matched groups (*n* = 10) [25], demonstrating the difficult, time-consuming and high-cost nature of this method.

Additionally, it should be noted that other tooth parameters should be taken into count, which cannot be sufficiently standardized by these methods, such as the age and ethnicity of donor and environmental factors. These factors have an impact on the mechanical properties of dentin [23,47,48].

Dentin is a hard tissue that occupies the majority of the human tooth, which possesses a complicated hierarchical structure. It consists of approximately 45% mineral material, 33% organic material and 22% water [48,49].

The microstructure of dentin is largely dominated by its tubules, which extend radially outward from the pulp towards the dentin–enamel junction. The lumen of each tubule is surrounded by a cuff of peritubular dentin, which consists of a highly mineralized region of apatite crystals and a small proportion of organic proteins. The tissue located between the tubules is called intertubular dentin and contains a matrix of collagen fibers reinforced by apatite [49,50].

After the third decade of life, there is a transition in the microstructure of dentin, in which the inorganic material increases and tubules become gradually filled with it [48,49,50,51], consequently diminishing the tubule density and dentinal diameter. These modifications produce variations in its mechanical properties, such as an increase in elastic modulus and hardness and a decrease in strength and fatigue crack resistance [49,50,51,52,53]. It has been well established that the aging of dentin starts from root dentin and continues in the coronal direction [49,54]. The occlusion rate of dentinal tubules is greatest at the root apex and closer to the pulp in older donors, when compared with younger donors, where it is greater near the cementum [54].

Additionally, in a study of the microstructure of dentin and its mechanical behavior, existing differences were demonstrated between the dentin of donors with an origin in the United States of America from those with an origin in Colombia [47].

For the reasons listed above, a total standardization is, with actual methods, almost impossible to achieve.

Three-dimensional-printed teeth that reproduce the features of natural teeth may overcome all these limitations [19,28,31,34] and are also suitable for practicing access opening, canal instrumentation, radiographic length control and canal filling [26,28].

(1) Difficult to collect: There is no problem in collecting them since the selection of different teeth is not limited and they are available immediately in a sufficient number [19,23,26,28,44]. Additionally, an online platform where institutions could share their printable files would be a considerable benefit for the entire community [26].
(2) Ethical considerations: Only 3D-printed teeth generated from cone beam computer tomography (CBCT) or micro-CT of natural teeth should require donor consent for the use of their teeth [23,28,32]; however, the number of natural teeth needed would be diminutive.
(3) Potential cross-infection risk and storage: They present no risk, are more hygienic and have better handling since they do not need to be stored in liquid, providing a safe training environment [19,44,55].
(4) Standardization: They are realistic and standardized, so the same level of difficulty is guaranteed for every single student and, consequently, they will be scored fairly [19,26,27,28,44,56], because this enables the definition and standardization of specific grading criteria [30,57]. This standardization allows students and instructors to focus on learning and teaching the clinical procedures, rather than dealing with the morphological variability of natural teeth [24]. Additionally, in ex vivo studies, this morphological standardization has a major impact on the credibility of results [2,25,33,58,59].

Three-dimensional-printed teeth have other advantages in pre-clinical courses such as presenting canal anatomy difficulty in a progressive way [2,19]; students can practice the procedures as many times as they want and even compare different protocols, which is also true for ex vivo studies [2,19,35]. It seems logical that techniques well covered in pre-clinical course are performed more easily and cause less stress among the students when treating patients for the first time [16].

Tchorz et al. 2015 compared the effect of students training with artificial teeth and with extracted human teeth on the quality of the first root canal treatment performed on patients. They were unable to detect any differences between the two groups [37], demonstrating that artificial teeth are suitable for endodontic training.

Various studies report that students highly appreciated the use of 3D-printed teeth for the standardization of endodontic training and as a realistic simulation of clinical treatment [26,34,55]. Moreover, instructors appreciated 3D-printed teeth, possibly because they also experience the difficulties of pre-clinical courses on natural teeth [27,30].

Another use for 3D-printed teeth is in complex anatomical clinical cases, allowing clinicians to understand the root canal configuration and try a specific clinical treatment before reproducing it on a patient. The clinician can train and find the best approach to be more confident during the real treatment [1,60,61].

## 3. How

**Figure 1:**
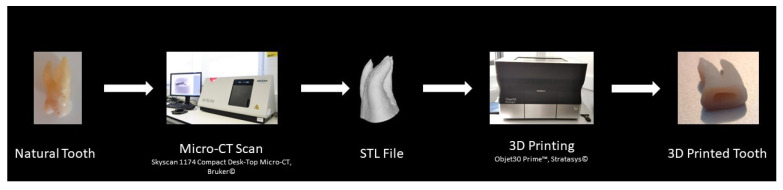
Three-dimensional printing process. (Micro-CT—Micro-Computer Tomography; STL—Standard Tessellation Language).

Additive manufacturing, also known as 3D printing, is the process of joining materials to make parts from 3D model data, usually layer upon layer [62]. This technology relies on 3D data, post-processing tools and algorithms to restructure and edit multiple planes to form a 3D model and ultimately generate a part using a specific material with a 3D printer [1,59,63] (Figure 1).

The first step of 3D printing is to obtain a digital model based on computer tomography (CT) scan data, laser scan data, designed using computer-aided design (CAD) [63,64].

Micro-CT is a non-invasive, non-destructive and high-resolution technology that allows the three-dimensional study of the root canal system. This technology can be used to understand the influence of different procedures on the canals by digitally reconstructing cross-sections of the teeth, which can be grouped to create three-dimensional models [65]. Micro-CT outputs images in Tagged Image File Format (TIFF). These TIFF images are reconstructed in Bitmap (BMP) format and are then managed into 3D construction software to output data in STL format [59,66].

CBCT is a modification of the CT concept, involving the single rotation of an X-ray source around the subject. CBCT outputs images in Digital Imaging and Communications in Medicine (DICOM) format, which are then managed into 3D construction software to output data in STL format [58,63,67].

STL format stands for Standard Tessellation Language or Standard Triangulation Language and represents the virtual 3D surface with the help of triangulation (tessellation). Each triangular face is characterized by its three corner points and the corresponding surface. Curved surfaces are approximated polyhedral. Increasing the number of polyhedral, by diminishing the size of the triangles, describes the object surface with higher resolution; however, this exponentially increases the number of triangles in the file, which results in large file sizes [36,68].

The accuracy of 3D printers is defined by XY resolution and layer thickness, namely the z-axis, which represents the vertical accuracy. The layer thickness typically describes the surface finish, so a thinner layer thickness is associated with smoother objects but also with a longer printing time [36,69].

In some 3D printing processes, areas under overhangs, cavities and holes are filled with a support material, and its removal is dependent on the technology used [36].

The various additive processes of major interest for this aim are presented.

Stereolithography (SLA) (layer thickness 25–100 µm) polymerizes by a single ultraviolet (UV) laser, a bath of liquid resin, which is held in a vat [31,36,64,69,70]. A computer-controlled mirror is used to focus the UV laser onto the surface of the resin and cure the resin layer by layer. During the sequential curing process, the layers bind together to form a solid mass, beginning from the bottom of the object and building upward; this is the bottom-up approach [36,69,70]. In contrast, in the top-down approach, the platform can be lowered vertically and is immersed in the reservoir. The resin layer is exposed to a laser that scans from the bottom reservoir [36]. The model is then removed and post-cured for a further period in a UV cabinet for a reduction in the residual resin content [36,70]. The model material is robust, slightly brittle and relatively light [70]. It requires support material [36].

Digital Light Processing (DLP) (layer thickness 25–100 µm) polymerizes by a light source from a conventional DLP projector. Liquid resin is held in a vat [31,36,70]. The projector exposes the resin to a 2D image and the object is produced by layers as the platform is manipulated [69], which is similar to SLA, but there is no moving beam [63]. It requires support material [36].

Fused Deposition Modelling (FDM) (layer thickness 178 or 254 µm) is a method in which layers of molten material are deposited from a filamentous nozzle and then solidify within 0.1 s. Generally, it is less accurate [36,64,69,70,71]. It may need support material [36].

PolyJet and MultiJet printing technology (layer thickness up to 16 µm) uses photosensitive resin to provide highly accurate product detail, is not limited by geometric shapes and is particularly suitable to simulate root canal anatomy. This method is very similar to the 2D printing process of an inkjet printer [1,36,44,55,64,70]. This technology prints two different materials within a single print job: a model material and the support material [36,44,63,70,71]. The models are created by the jetting of ultra-thin layers of resin that are cured with UV light after each pass onto a build tray that lowers and moves backward and forward. In these technologies, the support material is softer [44,64,69,70,71,72]. Typical removal methods include manual breaking, dissolution in water pressure, dissolution in caustic soda or melting by raising the temperature [36,44,72]. These two printing technologies only differ in their support materials and post-processing requirements [36,69,70].

ColorJet printing technology (layer thickness 50–100 µm) is a method in which a print head selectively disperses a binding agent onto layers of powder that are glued together [36,64,69,70]; however, the accuracy, finish and strength are poor [63]. In this method, the support material is not needed because the surrounding powder supports the unconnected parts, and in the finishing process, the prototype is infiltrated with a cyanoacrylate-based material to harden the structure and diminish the porosity [36,70]. It does not require support material [36].

Selective Laser Sintering (SLS) (layer thickness 30–100 µm) and Selective Laser Melting (SLM) (layer thickness 20–100 µm) printers use a computer-directed laser and a roller to distribute layers of powdered material on top of the preceding layer; each new layer is sintered (SLS) or melted (SLM) [36,64,69,70], and they can print a thermoplastic polymer, metal, ceramic or glass [36,63]. They do not require support material [36].

## 4. Problems

Three-dimensional-printed teeth still present some problems that need to be overcome before they can be used routinely in studies or in education.

Three-dimensional-printed teeth can be used only for studies on the root canal shape; they are not suited for root canal cleanliness or alterations in dentin [23], although, recently, Choi et al. 2021 modified a designed 3D-printed tooth with a slot wherein a dentin segment could be inserted. After bacterial contamination of the dentin, various protocols of irrigation were applied and compared [29]; however, more studies are needed with this prototype. Nevertheless, they could be used in obturation studies, Karatekin et al. 2019 and Gok et al. 2017 evaluated different obturation techniques in 3D-printed teeth with C-shape canals, by cross-section at different levels and analyzing the images with magnification [58,73]. In the study by Karatekin et al., 3D-printed teeth were instrumented, and as the authors stated, this could be a limitation of their study, since one major advantage of 3D-printed teeth is standardization and the bias introduced by instrumentation was not considered. In the study by Gok et al., the natural tooth was instrumented prior to the micro-CT scan; this methodology is more adequate for obturation studies. Additionally, Peters et al. 2021 used 3D-printed teeth obtained from a previously prepared tooth to compare the obturation quality between students [74].

It has also been reported that it is difficult to remove the support material from parts with features with fine details [23,26,45,72]. To the author’s knowledge, there is no study that demonstrates that the internal root canal anatomy is free of support material, and the majority do not explain the protocol used for removing it from inside the root canals, or even if it was performed [1,55,58,59,66,73]. This way, canals could be filled partially or totally with the support material. The effect of this material on the variables of interest should be considered. Reymus et al. 2019 described the method used in their study for removing the support material. First, on the natural tooth before a CBCT scan, they carried out a retrograde preparation on the apical 5 mm with hand files up to an ISO file 10 to ensure patency, and in sequence, all canals were prepared up to an ISO file 20. Subsequently, the access cavity was closed with a cotton pellet and a radiopaque filling. After being printed, using the SLA technique, the 3D-printed teeth were cleaned with alcohol for 2 min in an ultrasonic bath. Next, they were centrifugated for 10 min at 3500 revolutions per minute to free the canals from the support material [26]. The authors stated that for support material removal, the canal must be patent, and that is the reason for executing the retrograde preparation [26]; however, with this protocol, the internal anatomy of the natural tooth was altered and did not mimic an intricate anatomy. Orel et al., 2021, in a study comparing different instrumentation systems, reported that the apical 3 mm of the canals of 3D-printed teeth, using the SLA technique, were obstructed by the support material, so they manufactured another 3D-printed tooth separated along the long axis of the tooth, into two pieces that were held together with a clamp [45]. Since they were comparing the effects of different instrumentation systems on the canal walls, the fact that the teeth were separated and only held together by a clam during the process could have possible effects on the variables of interest that were not addressed by the authors.

Some studies analyzed the accuracy of 3D-printed teeth by analyzing the external surface [1,26,75], and others, the internal root anatomy [1,59,66].

Reymus et al. 2019 only analyzed the outer surface of the 3D-printed teeth by comparing the STL file of the natural tooth, with STL files of the 3D-printed teeth obtained with a dental scan, and stated that could be assumed that the results would be similar for the internal root anatomy [26], but they did not carry out any evaluation of this parameter.

Liang et al. 2018 compared, by CBCT, the accuracy of the external surface and the internal root anatomy at 3, 6 and 9 mm from the apex, between twenty natural premolars and their respective 3D-printed teeth. It was concluded that 3D-printed teeth did not differ significantly from the natural teeth [1]. It is important to mention that the natural teeth were previously divided into four groups and instrumented previously to 25#06, 30#09, 35#04 and 40#40, respectively, so the results cannot be extrapolated to teeth that would be used in studies or education with intricate canal anatomy.

To the author’s knowledge, only one study to date has used micro-CT to compare the internal root anatomy of the natural tooth with the internal root anatomy of 3D-printed teeth [66]. Cui et al. 2018 scanned six pre-molar teeth, three maxillary and three mandibular, and used these scans to construct 3D-printed teeth using the PolyJet technique, removing the support material by melting. However, the protocol was not explained, and it is not noted whether the 3D-printed teeth had an access cavity carried out on them. They concluded that the area and volume of the natural canals were not significantly different from the printed canals [66]. However, for the validation of their model, only one 3D-printed tooth was compared with the respective natural tooth, creating six experimental pairs. So, with this methodology and with such a small number of 3D-printed teeth, it cannot be concluded that the 3D printing of the internal root anatomy is consistent when the same tooth is printed on a large scale.

Xu et al. 2021 evaluated the shaping ability of four single-file systems on 3D-printed teeth, using the PolyJet technique. Micro-CT scans were executed before and after instrumentation for each specimen [59]. One issue with this methodology is that, since the 3D-printed teeth were not compared with the natural teeth, the consistency of the printing technique cannot be assumed, and the pre- and post-instrumentation micro-CT scans continue to be needed. One objective of 3D-printed teeth is also the diminishing time and costs, which was not accomplished in this study.

The major concern is the difference in radiopacity and hardness between the resin and dentin as referred in most studies [2,23,26,55,58]. In the study of Reymus et al. 2019, students considered that the preparation of 3D-printed teeth was easier than the preparation than the natural tooth as a consequence of this difference in hardness [26]. However, it should be noted that the natural tooth was prepared upan ISO file 20 before the scan to obtain the STL file which could also have contributed to an easier preparation by the students.

A recent study by Cresswell-Boyes et al. 2022 compared 3D-printed typodont teeth with materials developed to mimic both the morphology and mechanical response of natural teeth. Their results indicate that 3D-printed typodont teeth replicating enamel and dentine can be mechanically comparable to extracted human teeth despite the material compositions differing from the materials found in human teeth [76]. Nonetheless, they used a 50 µm layer printer and only printed a typodont tooth without internal anatomy; however, these results are promising but more studies are needed in order to determine if these materials could be used in future 3D-printed teeth endodontic studies.

Another concern is, since the minimum layer thickness of 3D printers is 16 µm, the initial diameter of the root canal of the natural teeth to be printed cannot be smaller than ISO-size 15 [23], but if taken into consideration that most apical foramen diameters vary between 0.20 and 0.29 mm [45], this is perhaps not an issue. However, smaller structures such as lateral canals and narrow isthmuses probably cannot be reproduced [26,56].

The process of making 3D-printed teeth involves various steps, each of which can be a source of error [75]. For developing 3D-printed teeth, a variety of powerful freeware and open-source software solutions are available [31,66,75]; once more, the lack of standardization and the absence of protocols promote different methods of measurement and accuracy of studies making it impossible to compare the results of different studies [66].

**Figure 2:**
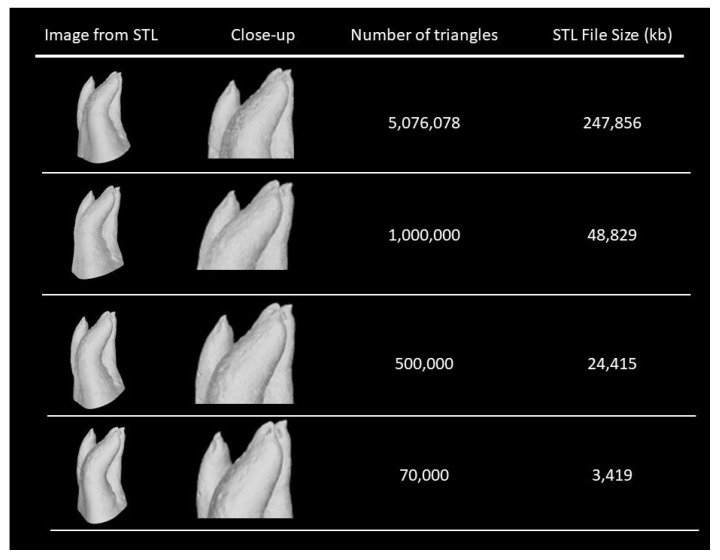
Differences between the number of triangles and size in kilobytes (Kb) of Standard Tessellation Language (STL) files of the same maxillary first molar.

Another question is the resolution of the scan needed for obtaining high-quality 3D-printed teeth. Kulczyk et al. 2019 compared the external surface of 3D-printed teeth obtained by different methods of 3D data acquisition, namely by CBCT, optical 3D scanning and micro-CT. They concluded that even though CBCT seems to be a sufficient method to obtain data for 3D-printed teeth, it does not equal micro-CT. They scanned a canine and a molar, and the number of triangles of the data collected by CBCT in high resolution were 65,340 and 98,624 for each tooth, respectively. With micro-CT, the numbers were 11,510,788 and 24,974,112 for each tooth, respectively. As stated before, the higher the resolution of the original data, the better the quality of the 3D model and the better the quality of the 3D-printed object. Nonetheless, 3D models created based on micro-CT data provide better accuracy than 3D printers have been able to produce to date [75]. Additionally, a very-high-resolution STL creates an excessively large file that is difficult for 3D printing software to process (Figure 2).

## 5. Future

As previously stated, the main goal of using 3D-printed teeth is standardization. For the scientific community, this means that the possibility of creating equal protocols for ex vivo studies could allow an easy comparation of results. For the teaching community, the fact that students all have the same object of training could allow a fair evaluation and an equal progression, since every student would have the same tooth with the same degree of difficulty according to the teaching stage. We should keep in mind that for 3D-printed teeth to make any sense, it is necessary to print these teeth on a large scale, rapidly and with low associated costs.

So, to achieve the degree of standardization needed and the establishment of protocols, it is the author’s opinion that the problems with 3D-printed teeth presented here should first be answered in this order, and they are summarized in Table 1.

(1) From the great plethora of existing commercial printing materials, it should be established which materials are more dentin-like. Through the fabricant datasheet, we can choose the potential materials available and compare their physical and mechanical properties and radiopacity with human dentin. This way, subsequent studies will only use the material most appropriate for each printing technique.
(2) The establishment and validation of a protocol, for each printing technique, of the total removal of the support material in 3D-printed teeth with unaltered canal anatomy could be easily achieved if the 3D-printed teeth have an access cavity already created. In sequence, establishment is the printing technique that produces consistent 3D-printed teeth with the best accuracy by comparing the internal anatomy of the natural tooth with a large number of 3D-printed teeth by using micro-CT.
(3) Investigate whether the support material influences the instrumentation technique outcomes. This could be performed by comparing teeth in which the support material was removed with teeth where the support material was not removed. If the support material does not influence the variables of interest, the support material could probably mimic the pulp tissue and could permit the printing of a 3D tooth with a closed crown and an unaltered internal root anatomy.
(4) Evaluate the minimum and maximum number of triangles in STL files needed for maintaining the accuracy of the 3D-printed teeth. From the literature review, it is known that the higher the resolution of the original data, the better the quality of the 3D model and the better the quality of the 3D-printed object. In fact, we do not know if the large number of triangles obtained by micro-CT is necessary to achieve the necessary accuracy of the canal anatomy. Resources are perhaps being overused, since, as stated before, a very-high-resolution STL file is difficult for a 3D printing software to process. Additionally, it is necessary to establish well-defined and well-described step-by-step protocols for a reasonable number of software programs needed for the entire process of 3D printing. These protocols would eliminate a great number of confounding factors since errors can be introduced during image acquisition, image segmentation and 3D image construction.
(5) After addressing all the problems with the printing techniques, an ideal 3D printing material containing hydroxyapatite or ceramic particles that can mimic the radiopacity and/or hardness of human dentin could be developed.

**Table 1:** Recommendations for future research of 3D-printed teeth in endodontics.

Problems	Future
Differences in radiopacity and hardness between resin and dentin	Establish from the commercial printing material which of them are more dentin-like Develop an ideal 3D printing material containing hydroxyapatite that could mimic human dentin
Removing support materials from root canal system Validation of printing accuracy of internal anatomy	Establishment and validation of a protocol for the total removal of support material from root canal system Investigate the influence of support material in the instrumentation technique outcome
Variety of powerful freeware and open-source software solutions available	Establish well-defined and well-described step-by-step protocols for a reasonable number of software programs
Resolution of STL files and size	Evaluate what is the minimum and maximum number of triangles in STL files needed for maintaining the accuracy of 3D-printed teeth

STL—Standard Tessellation Language.

## 6. Conclusions

Three-dimensional-printed teeth may become the gold standard in ex vivo studies and in endodontic training, substituting natural teeth. Nonetheless, to realize this, more studies are needed to resolve the problems that 3D-printed teeth still present. Additionally, more efforts should be made to develop well-established protocols so the standardization of methods can be achieved, and then the results can be compared between studies.

## Data Availability

Not applicable.
